# Exploring caregiver experiences and support needs in end-of-life care for people living with HIV: A scoping review protocol

**DOI:** 10.1371/journal.pone.0313879

**Published:** 2024-12-02

**Authors:** Kristina M. Kokorelias, Andrew D. Eaton, Marina Wasilewski, Tyler Redublo, Luxey Sirisegaram

**Affiliations:** 1 Section of Geriatric Medicine, Department of Medicine, Sinai Health System and University Health Network, Toronto, Toronto, Ontario, Canada; 2 Department of Occupational Science & Occupational Therapy, Temerty Faculty of Medicine, University of Toronto, Toronto, Ontario, Canada; 3 National Institute on Ageing, Toronto Metropolitan University, Toronto, Ontario, Canada; 4 Rehabilitation Sciences Institute, Temerty Faculty of Medicine, University of Toronto, Toronto, Ontario, Canada; 5 Faculty of Social Work—Saskatoon Campus, University of Regina, Saskatoon, Canada; 6 St. John’s Rehab, Sunnybrook Research Institute, Toronto, Ontario, Canada; 7 Research & Academics, Ontario Shores Centre for Mental Health Sciences, Whitby, Ontario, Canada; University of Cape Town, SOUTH AFRICA

## Abstract

**Background and objectives:**

End-of-life care supports individuals in the last few weeks or months of their life and their caregivers, offering psychosocial support, symptom management and relief, and resources. While some of the first public end-of-life care facilities were established due to HIV/AIDS, the current needs of caregivers for people living with end-stage HIV are not well understood. Caregivers provide two-thirds of the care for people living with HIV, yet their specific support needs and experiences are under-researched. Existing strategies often use a “one-size-fits-all” approach, which may not address the unique challenges faced by these caregivers, such as stigma and lack of social support. This study aims to synthesize the literature on the end-of-life care experiences and needs of caregivers for individuals living with HIV.

**Research design and methods:**

A scoping review, guided by Arksey and O’Malley’s framework and the Joanna Briggs Institute’s recommendations, will be conducted. An Information Specialist will assist in developing a search strategy to be applied across databases like Medline, Embase, PsycINFO, and PubMed. Search results from each database will be imported into Covidence software for duplicate removal and title and abstract screening. Two researchers will independently screen studies using the ‘Population–Concept–Context’ (PCC) framework, with screening conducted at two levels: title and abstract, and full-text. The inclusion criteria will be piloted on a random sample of articles to ensure inter-rater agreement (kappa statistic >0.61). Disagreements will be resolved through discussion or with the involvement of a content expert if needed. Final selections will be reported using the PRISMA flow diagram, and reasons for exclusion will be documented.

**Discussion and implications:**

The findings from this scoping review will provide valuable insights into the end-of-life care experiences and support needs of caregivers for individuals living with HIV. By identifying common themes and challenges, such as caregiver fatigue, emotional strain, stigma, and lack of social support, this study will underscore the inadequacy of the current “one-size-fits-all” approach in addressing the unique needs of these caregivers. This research has the potential to influence both clinical practice and policy by advocating for more personalized support strategies within end-of-life care settings.

## Introduction

### Rationale

During the height of the Human Immunodeficiency Virus (HIV) and Acquired Immune Deficiency Syndrome (AIDS) epidemic in the 1980s and 1990s, end-of-life care became synonymous with AIDS care due to the high mortality rates and the severe, progressive nature of the illness at that time [1, 2]. HIV is a virus that attacks the body’s immune system, specifically targeting CD4 cells (T cells), which are crucial for immune defense [1, 2]. If left untreated, HIV can weaken the immune system to the point where it becomes unable to fight off infections and diseases. AIDs is the most severe phase of HIV infection, occurring when the immune system is significantly damaged, leading to life-threatening infections or cancers [3]. While there is no cure for HIV, antiretroviral therapy (ART) can manage the virus and prevent the progression to AIDS, enabling individuals to live longer, healthier lives [3].

Many of the world’s first hospices opened due to the AIDS crisis. Care models were heavily focused on managing complex symptoms, providing psychological support, and addressing the profound stigma associated with living with HIV [4, 5]. End-of-life care provides crucial support not only to individuals facing death but also to their caregivers, and entails essential psychosocial support (e.g., grief counseling), symptom management/relief (e.g., mental discomfort reduction, pain medication), and provision of necessary resources [6–8]. End-of-life care may be synonymous with hospice care and can be a form of palliative care.

With the ART and other medical advancements, HIV has transformed from a terminal illness to a manageable, chronic condition for many [1, 2]. In fact, many HIV-positive individuals now reach end-of-life without reaching the clinical criteria for AIDS [9]. In other words, many people living with HIV will die from a non-AIDS-defining illness. This shift has fundamentally altered the landscape of HIV care, including end-of-life care needs. The once inevitable progression to HIV-related death has become less common, resulting in fewer experiences of end-of-life care specific to HIV in clinical practice [10, 11]. Consequently, the experiences and support needs of caregivers for people living with HIV who do reach an advanced, end-stage illness and are seeking end-of-life care remain underexplored and less well understood [12, 13].

Family caregivers, often family members or close friends, played a pivotal role in providing daily care and emotional support during the HIV epidemic [14]. Here within we define ‘caregivers’ as family members, neighbors or close friends who provide unpaid care to persons living with HIV. Family or personal caregivers continue to provide approximately two-thirds of all care for individuals living with HIV undergoing end-of-life care [15]. Despite their significant contributions, there is a notable gap in the literature regarding the specific support needs and end-of-life care experiences of these caregivers [16, 17]. The existing literature suggests that caregiving at the end of life for individuals living with HIV can result in caregiver fatigue, emotional and physical strain, and significant financial burden [12]. Traditional caregiver support strategies in end-of-life care, often based on a "one-size-fits-all" model [14], may not adequately address the unique challenges faced by caregivers of people living with HIV, such as enduring stigma and limited social support [18–21]. Recognizing these distinct challenges is essential for optimizing the well-being and effectiveness of HIV caregivers within end-of-life care. Tailored support interventions that consider the unique context of HIV caregiving are needed to better address these caregivers’ needs.

### Objectives

As more individuals with HIV are dying from non-AIDS-related issues, caregivers are encountering new and complex challenges in providing end-of-life care and support for this population. Research is needed to better understand these evolving experiences and address the specific needs and barriers faced by caregivers in this context. To date, there has been no comprehensive synthesis of the available evidence on the end-of-life care experiences and support needs of caregivers for individuals living with HIV. This scoping review aims to fill this gap by systematically examining and synthesizing the published literature, providing a foundational understanding that can guide future research, policy, and clinical practice in this area. Thus, the primary goal of this scoping review is to understand the current state of end-of-life care experiences and needs among caregivers of individuals living with HIV.

The review will explore various aspects of HIV end-of-life care, including caregiver challenges, support strategies, and tailored interventions. Specific questions guiding this review include:

1. What does end-of-life care provided by caregivers of people living with HIV entail?
2. What are the unique challenges faced by caregivers of people living with HIV in end-of-life care?
3. What interventions and/or support strategies exist for these caregivers?

## Methods

### Design

The preparation and development of this protocol followed the guidelines outlined in the Preferred Reporting Items for Systematic Reviews and Meta-Analyses for Protocols 2015 (PRISMA-P 2015) [22] **(S1 Checklist)**. This scoping review protocol was developed in alignment with the Joanna Briggs Institute (JBI) guidelines [23, 24] and follows the framework proposed by Arksey and O’Malley [25]. The framework consists of five stages: (1) defining the research questions, (2) identifying relevant studies, (3) selecting eligible studies, (4) charting the data, and (5) collating, summarizing, and reporting the results [25]. The review will be conducted and reported according to the PRISMA extension for Scoping Reviews (PRISMA-ScR) checklist [26].

This protocol has been preregistered on the Open Science Framework (OSF) platform and is accessible via OSF Registries at: (Blinded for Review). There will be no direct involvement of patients or the public in this review. Since this review involves the analysis of previously published literature and does not include human participants, ethical approval is not required.

### Positionality

Positionality statements enhance transparency and rigor by recognizing how the research team’s background may influence the research [27]. This mixed-gender research team is made up of Canadian researchers with experience in the study of HIV and in the study of caregiver experiences. Members include MSc and PhD-trained researchers and clinicians (social work and internal and geriatric medicine) with various ethnic identities. This research is motivated by the research team’s own interest in supporting family-centered models of care [28].

### Identifying relevant studies

To obtain a comprehensive overview of existing data, we chose databases with both multidisciplinary and specialized scopes.

For this scoping review, we will search the following seven databases to identify and gather relevant literature: MEDLINE (Ovid), CINAHL, Embase, APA PsycNet®, CINAHL® Plus, ERIC, Scopus™, and the Web of Science™ Core Collection. With feedback from the research team, a health service librarian will create a rigorous search strategy that will be tailored to each relevant database. The search will be conducted in January 2025 by an information specialist [29] from the inception of the journal until 2025. Additionally, secondary searches will be carried out by reviewing the reference lists of included studies and relevant systematic reviews. No restrictions will be placed on language or publication date, considering the evolution of HIV treatment and its impact on palliative care.

To ensure the search strategy effectively identifies relevant articles in the databases, we will utilize the evidence-based checklist from the Peer Review of Electronic Search Strategies 2015 (PRESS 2015) guideline [30]. The search strategy will be reviewed by another health-service librarian.

Grey literature will not be reviewed due to the difficulty in ensuring the reliability and comprehensiveness of non-peer-reviewed sources [31].

### Selecting eligible studies

Search results from each database will be imported into Covidence software to remove duplicates and facilitate title and abstract screening [32]. Two researchers will independently screen the studies using the ‘Population–Concept–Context’ (PCC) framework [33]. Articles will be reviewed at two levels: title and abstract screening and full text screening. Before screening titles and abstracts, the inclusion criteria will be piloted on a random sample of articles (~5) to ensure that the minimum inter-rater agreement among reviewers is achieved (i.e., kappa statistic >0.61) [34, 35]. **Table 1** outlines the inclusion and exclusion criteria. If needed, the inclusion criteria will be adjusted to enhance clarity [25]. Disagreements in both levels of screening (i.e., title and abstract and full text) will be resolved through discussion between the two reviewers. When deemed necessary, a content expert (clinician with expertise in HIV care) will be invited as a third reviewer to make the ultimate decision (LS). Full-text articles will be reviewed to determine eligibility, and the final selection will be reported using the PRISMA flow diagram [26]. Reasons for excluded studies will be recorded and reported. To ensure comprehensive data collection, we will also reach out to authors of relevant studies to identify any additional sources, including unpublished or in-progress research that may contribute to the review.

**Table 1:** Inclusion and exclusion criteria.

Criteria	Inclusion	Exclusion
**Population/Participants**	Unpaid individuals who provide care to persons living with HIV who are also experiencing palliative and/or end-of-life care.	Individuals who do not have an active role in providing care to persons living with HIV.
**Context/Intervention**	Any context or intervention specifically designed to support caregivers of individuals living with HIV, including emotional, psychological, and social support programs. Interventions that involve end-of-life care approaches to persons living with HIV where caregivers are involved in the care process. This may include pain management, symptom control, and end-of-life care strategies where caregiver involvement is crucial.	Contexts or interventions that do not directly involve or target caregivers, such as those focused solely on patient treatment without considering caregiver needs. Programs or strategies that target a broad population without a specific focus on caregivers of individuals living with HIV. Interventions that do not include or consider the end-of-life care aspect of caregiving for individuals living with advanced HIV.
**Concepts**	Focus is on end-of-life care: A multidisciplinary approach that provides relief from the symptoms and stress of a serious illness, focusing on improving the quality of life for both the patient and the caregiver [36].	
**Types of sources**		
**Design/date**	Any empirical study design, any date	Non-empirical literature (e.g., scoping reviews) and relevant grey literature (e.g., conference abstracts, theses and dissertations)
**Language**	English	While our search will impose no restrictions on language, we will only include studies published in English or full text will be excluded. The inclusion of "no language restrictions" in the search strategy is aimed at ensuring comprehensiveness by capturing all potentially relevant studies across multiple languages. By not limiting the search to English-only terms or sources, the authors broaden the initial search scope, which reduces the risk of missing critical research conducted in non-English-speaking regions. This approach allows the identification of all relevant studies and adds transparency by documenting the presence of non-English studies, even if these are ultimately excluded due to language barriers at the full-text review stage.

### Charting the data

A data extraction form, adapted from the JBI template [23], will be used to chart relevant information from the selected studies. The research team will collaboratively develop the charting form. The charting form will include several key data elements to ensure comprehensive data collection. First, country will be recorded to capture the geographical context of each study, which will help us understand any regional variations in caregiver experiences. The date and year of publication will also be noted to assess the recentness of findings and potential evolution of caregiver needs over time.

Data on participant details will include age, which allows for an analysis of age-related differences in caregiving experiences; gender, to examine how caregiving roles and experiences might differ by gender; and ethnicity, which may reveal cultural factors influencing caregiving practices and challenges.

We will record specific caregiver challenges identified in the literature, including emotional burden (such as stress, anxiety, and grief related to caregiving), financial burden (covering the economic strain or income loss associated with caregiving), and physical burden (encompassing the physical health impacts of caregiving, such as fatigue and other physical stressors).

The charting form will also capture support strategies used by caregivers. This includes formal support systems (such as professional healthcare services, counseling, and respite care) and informal support systems (support from family, friends, and peer networks).

Lastly, the form will include data on the impact of stigma on caregivers, focusing on social isolation (the sense of separation from community due to HIV stigma) and discrimination (any prejudicial treatment caregivers may experience within healthcare settings or the wider community). These variables are essential to provide a comprehensive overview of the support needs and challenges.

#### Risk of bias assessment

Articles will be appraised using the Critical Appraisal Skills Program (CASP) [37]. The CASP is a widely used tool for evaluating various types of research studies, including qualitative ones within scoping reviews [37]. The CASP checklist assesses the risk of bias in qualitative research through three key questions: "Are the study results valid?" (Section A), "What are the results?" (Section B), and "Will the results be helpful locally?" (Section C) [37]. It includes 10 questions across these sections, with nine rated as "yes" (2 points), "can’t tell" (1 point), or "no" (0 points), totaling a maximum score of 18 [37]. The 10th question requires a qualitative answer [37]. Studies will be rated on a three-star system by two reviewers: low (one star; 0–6 points), moderate (two stars; 7–12 points), or high (three stars; 13–18 points). The two reviewers’ voting will be compared and discussion will occur through research team discussion until consensus is met. A summary table will present these CASP ratings.

### Data analysis

The charted data will be collated to provide a comprehensive overview of the current state of end-of-life care for caregivers of individuals living with HIV. The findings will be presented through a narrative summary, supported by tables and diagrams. Qualitative data will be analyzed using thematic analysis [38, 39], with the charted data being used to assist in the process. The research team will collaborate to explore and discuss emerging themes and concepts. To do so, two team members will independently code data from the included studies using a line-by-line approach [40], with codes subsequently compared and consolidated through discussion to develop a comprehensive codebook [40]. This codebook will then guide the coding process for the remaining data. Following coding, higher-level descriptive and analytical themes will be derived by clustering similar or contrasting codes [40]. Regular team meetings will facilitate peer debriefing and analysis auditing [41]. Team meetings will include identifying key challenges, existing support strategies, and areas where tailored interventions are needed. The results will emphasize key themes, quotes, and interpretations. Given the challenges of qualitative data synthesis, detailed documentation of the analytical process will be maintained, including the rationale behind decisions, using methods such as mind mapping and charting, to ensure clarity and transparency throughout the review [42]. Recommendations for future research, practice, and policy will be proposed throughout team discussions.

## Discussion

Despite the significant role of family caregivers in the care of people with HIV, there is a notable gap in understanding the challenges they face, including fatigue, emotional strain, and financial burden [12, 13]. The forthcoming scoping review aims to address this gap by systematically examining existing literature on the end-of-life care experiences and support needs of caregivers of those living with HIV. It will follow Arskey and O’Malley [25] and JBI guidelines [23], searching multiple databases with no restrictions on language or publication date. The review process includes rigorous screening, data extraction, and risk of bias assessment using the CASP tool. The findings will be summarized in narrative form, supported by tables and diagrams, with qualitative data analyzed thematically to provide recommendations for enhancing caregiver support in HIV end-of-life care.

### Summary of evidence

The forthcoming scoping review will make a substantial contribution to the field by filling a critical void in understanding the support needs and challenges encountered by family caregivers of individuals living with HIV undergoing end-of-life care. This scoping review will inform future research by providing a comprehensive overview of the current understanding of the end-of-life care experiences and support needs of caregivers for individuals living with HIV. Critically, this review will facilitate a reconceptualization of end-of-life HIV care by framing it away from AIDS-defining illnesses towards the new reality of mortality due to non-AIDS-defining illnesses. By systematically identifying and synthesizing existing literature, the review will highlight key gaps in knowledge, such as specific challenges faced by caregivers, effective support strategies, and the impact of stigma. This evidence base will serve as a foundation for designing targeted studies that address these gaps, offering insights into areas where further investigation is needed. The findings will guide researchers in developing more focused research questions and methodologies, particularly in exploring innovative support interventions tailored to the unique needs of caregivers of people living with HIV. Additionally, the review will reveal trends and patterns that can be used to refine existing models of caregiver support and identify best practices. Furthermore, the recommendations provided will inform the design of intervention studies and aimed at improving caregiver support.

### Limitations

This study protocol acknowledges several limitations. Firstly, the exclusion of grey literature, including conference abstracts and theses, may result in the omission of potentially relevant findings, which could limit the comprehensiveness of the review. Secondly, the protocol does not involve direct patient or public engagement, potentially missing insights from those directly affected by HIV caregiving. Additionally, the reliance on published literature means that any biases inherent in these studies could affect the overall findings. The selection of databases, while extensive, may also limit the scope if relevant studies are not indexed in the chosen sources. Another limitation is the restriction to English-language literature, which may introduce language bias. This restriction could limit the inclusion of relevant studies published in other languages, potentially narrowing the global perspective and diversity of caregiver experiences and support needs in end-of-life care for people living with HIV. Finally, the use of a single framework (i.e., CASP) for evaluating qualitative and quantitative studies may not fully capture the complexities of diverse research methodologies, potentially impacting the robustness of the conclusions drawn.

### Conclusion

In conclusion, this scoping review protocol represents a crucial step in advancing our understanding of the end-of-life care experiences and support needs of caregivers for individuals living with HIV. By systematically examining and synthesizing existing literature, this review aims to address the significant gaps in knowledge concerning the unique challenges faced by these caregivers. The findings are expected to reveal key insights into effective support strategies and highlight areas where tailored interventions are needed. This review will not only inform future research by identifying gaps and refining research questions but also guide the development of more personalized and comprehensive support strategies for caregivers. Despite the limitations of excluding grey literature and not engaging directly with patients or the public, the review’s rigorous methodology and adherence to established guidelines will provide valuable evidence to enhance caregiver support and improve end-of-life care practices. Ultimately, this work will contribute to a deeper understanding of the caregiving experience in HIV end-of-life care and foster the development of targeted interventions to better support this vital yet often overlooked aspect of care.

## Supporting information

S1 Checklist Preferred reporting items for systematic reviews and meta-analyses for protocols 2015. (PDF)
